# Proceedings of Mad River Valley Dental Society

**Published:** 1874-12

**Authors:** A. J. Grosvenor


### PROCEEDINGS OF MAD RIVER VALLEY DENTAL SOCIETY.

The Mad River Valley Dental Society met at the Philips House, Dayton, O., Oct. 15, 1874, at 10: 30 a. m. President Tizzard in the chair and the following members present: Drs. Tizzard, Watt, Berry, Runyon and Grosvenor.

There being just a quorum present the session was opened with prayer by Dr. Watt. Minutes of last meeting read and adopted. The Secretary reported what had been done by the Society toward the Barnum Testimonial, as follows:

Amount received as dues and arrears $12 00; amount received from Dr. French, society money in his hands $13 00; amount received from Dr. Stepp, society money in his hands, $7 00; Donated for society $2 00. Total from society $34 00.

Report read and adopted. Bill of $3 75 for printing, postage and stationery ordered paid. The executive committee's report was adopted as follows:

1. Histology of the teeth.

2. Diseases of the gums and their treatment.

3. Management of exposed pulps.

4. What are the requisites to secure success in filling teeth?

5. Smooth point pluggers, are they superior to serrated instruments?

5. Removal of superficial decay, will it in all cases effectually arrest the disease.

7. Best method of using the tooth brush, quality of brush and how often.

8. Flexible rubber disks.

9. What constitutes success in dental practice?

10 Dental education.

The President oppointed Drs. Keely and H. A. Smith committee on membership.

Dr. Tizzard presented a case of diseased antrum asking advice, etc. After some informal discussion adjourned to 2 p. m.

President Tizzard in the chair, 2 p. m., and the following members present: Dr. J. Taft, Watt, Berry, Corson, Bradley, Harlan, Keely, H. A. Smith, Tizzard, Runyan, J. C. Oldham, Boal and Grosvenor.

On motion of Dr. Keely, Drs. Jamison, of Connersville, Ind., and J. R. Clayton, of Shelbyville, Ind., and others that may be present were invited to participate in the discussions. The name of Dr. Whiteside, presented for membership and referred to the committee. On motion the first subject was temporarily passed, and the second taken up and opened by Dr. Taft, and continued at some length by Drs. Bradley, Corson, Watt, H. A. Smith and Tizzard. Subject passed and 6th. taken up, opened by a paper by Dr. H. A. Smith, also some extracts from an essay by Dr. Black, discussed until 5, when an adjournment was made until 7:30.

8 o'clock p. m. Reading of minutes dispensed with: 3d. subject opened by Dr. Taft and continued by others for some time, passed to 4th subject which was discussed at great length. 5th. opened by Dr. Bradley, the discussion was short, most of the members having but little experience.

Dr. Taft stated that he had just received a letter from Dr. Abram Robertson, author of an admirable work on extracting teeth, soliciting aid in the sale of his work, he being perfectly blind.

On motion, the Secretary was instructed that as soon as ten dollars were in the treasury it be presented to Dr. Robertson. Carried. It was suggested that all pay one dollar, which was responded to by several.

Dr. J. R. Clayton, President Indiana State Dental Society thanked the society for courtesies, and extended an invitation to all to attend the next session, stating that if the Secretary would send him a list of members he would see that all would receive circulars in time.

Membership committee recommended that Dr. Hubbard be made a member by paying one dollar and signing the constitution. Report adopted.

On motiod, adjurned to meet at Dayton, on the first Tuesday in April, 1875.

A. J. GROSVENOR, Secy.

